# The association between vertebral artery hypoplasia and fetal‐type variants of the posterior cerebral artery with imaging findings among patients with posterior circulation stroke: A single‐center cross‐sectional study

**DOI:** 10.1002/hsr2.1918

**Published:** 2024-02-22

**Authors:** Parvaneh Layegh, Lida Jarahi, Ehsan Hassannejad, Marziye Arab

**Affiliations:** ^1^ Department of Radiology, Faculty of Medicine Mashhad University of Medical Sciences Mashhad Iran; ^2^ Community Medicine Department, Faculty of Medicine Mashhad University of Medical Sciences Mashhad Iran; ^3^ Department of Radiology, School of Medicine Birjand University of Medical Sciences Birjand Iran; ^4^ Department of Radiology, School of Medicine Mashhad University of Medical Sciences Mashhad Iran

**Keywords:** fetal origin, posterior circulation, stroke, vertebral artery hypoplasia

## Abstract

**Background and Aim:**

The present study investigated the correlation between vertebral artery hypoplasia and fetal‐type variations of posterior cerebral arteries with stroke patterns and imaging findings in individuals with posterior circulation ischemic stroke.

**Methods:**

In this cross‐sectional study, patients with symptoms of acute ischemic stroke in the posterior circulation system referred to Ghaem Hospital in Mashhad between 2016 and 2022 were investigated. Demographic data, including age, gender, systemic diseases, history of previous stroke or transient ischemic attacks, and clinical manifestations of patients, were recorded using questionnaires and checklists from patient files. The results of imaging studies, including magnetic resonance imaging and computed tomography angiography, were also recorded. The obtained data were analyzed by SPSS statistical software.

**Results:**

Among 974 patients suffering from posterior circulation ischemic stroke, 155 patients with an average age of 60.44 ± 13.95 years were included in the study, out of which 97 patients (62.6%) were male. Unilateral vertebral artery hypoplasia on the right, left, and bilateral hypoplasia was present in 67 (43.2%), 35 (22.6%), and 5 (3.2%) patients, respectively. There were complete unilateral fetal origin on the right in 38 (24.5%), complete unilateral on the left in 12 (7.7%), partial unilateral on the right in 12 (7.7%), partial unilateral on the left in 6 (3.9%), complete bilateral in 14 (9%), and partial bilateral in 8 (5.2%) patients. There was no significant relationship between vertebral artery hypoplasia and PCA fetal‐type variants with different ischemia locations and infarct patterns (*p* > 0.05). Also, there was no significant relationship between the age and gender of patients with ischemia location and infarct pattern (*p* > 0.05).

**Conclusion:**

Despite previous evidence showing a relation between vertebral artery hypoplasia and PCA fetal‐type variants as risk factors for PC stroke, the present study did not establish a significant correlation between these factors and the location of ischemia and infarct patterns.

## 1 INTRODUCTION

Anatomically, the human brain consists of two segments with separate arterial systems. The posterior part of the brain is nourished by the left vertebral artery and the right vertebral artery.^1^, ^2^ A portion of the cerebral blood flow is provided by the vertebral arteries (VAs).^3^ The infratentorial structures of the brain (e.g., the cerebellum and the medulla) depend mainly on the VAs.^1^, ^3^ The VAs are originally divided from the first cervical part of the subclavian artery. In their course, these arteries travel medially toward the anterior scalene muscles before entering the transverse foramina. Following their passage through the foramen magnum, they merge and form the basilar artery (BA).^4^ These arteries are subject to a range of anatomical variants, such as asymmetry in the diameter of the two arteries. Despite the lack of a precise definition, vertebral artery hypoplasia (VAH) is usually characterized by a vertebral artery with an internal diameter equal to or less than 2 mm.^5^, ^6^ VAH was considered an innocent anatomical variation that would not lead to vertebrobasilar insufficiency (VBI).^7^, ^8^ Nevertheless, recent research has shed light on this subject, indicating that this condition may develop symptoms of VBI.^9^

Cerebrovascular diseases are a major cause of morbidity and mortality in neurological departments, resulting in a substantial financial burden.^10^ Posterior circulation (PC) stroke, classified as a subtype of stroke, occurs when a stroke develops within the structures supplied by the vertebrobasilar system. This includes the cervical vertebral arteries, as well as the intracranial vertebral, basilar, and posterior cerebral arteries.^3^, ^4^ PC and anterior circulation stroke differ in symptoms, signs, diagnostic tools, and treatment.^11^, ^12^ However, the risk factors associated with stroke in the PC are primarily identical to those of other cardiovascular conditions, including hypercholesterolemia, smoking, coronary artery disease, atrial fibrillation, and hypertension.^13^ Emboli originating from vertebrobasilar atherosclerosis or dissections are the main etiology for PC stroke.^14^, ^15^

Previous studies have shown that VAH is a risk factor for PC stroke.^7^ Additionally, regardless of the net flow, there is a positive correlation between the degree of VAH and the risk of a posterior ischemic event.^16^, ^17^ However, the literature has mainly discussed the effects of unilateral VAH rather than bilateral.^18^, ^19^ Moreover, studies have reported that the presence of a fetal‐type posterior circle of Willis is associated with VAH.^19^, ^20^ Therefore, we conducted the present study to investigate the relationship between imaging findings of patients with PC stroke and VAH and the fetal‐type variant of the posterior cerebral artery.

## 2 METHODS AND MATERIALS

The present study was a retrospective cross‐sectional study that included 974 patients with acute ischemic stroke in the posterior circulatory system. Those without a record of magnetic resonance imaging (MRI) or with inaccessible files were removed from the study. Finally, 155 patients diagnosed with an ischemic stroke at Gham Hospital from 2016 to 2022 were included in this study. Preliminary data, including gender and past medical history, were obtained from patient records. The location of ischemia, infarction pattern, the presence of VAH, fetal origin, and basilar artery hypoplasia (BAH) were recorded in a checklist. The patients had previously undergone a brain MRI and computed tomography angiography (CTA), which was extracted from hospital records. This study has been approved by the Ethics Committee of the Mashhad University of Medical Sciences.

### 2.1 Location of the ischemic stroke

MRI was employed to classify patients into distinct groups based on the location of cerebral ischemia. These groups included patients with an ischemic lesion in the proximal, middle, distal, and temporo‐occipital circulation. The presence of VAH was documented as left, right, or bilateral.

The identification and classification of infarction lesions based on vascular territory were performed using magnetic resonance imaging (MRI) and three‐dimensional time of flight (3D TOF) magnetic resonance angiography (MRA) examinations. A 1.5‐T MRI system from Siemens (Avento) was used with specific settings: T2‐weighted images with a TR/TE of 5000/91 ms, diffusion‐weighted images with a TR/TE of 3000/102 ms, and 3D TOF with a TR/TE of 31/7.86 ms. The locations of ischemic stroke were divided into four categories based on intracranial PC territories: proximal (medulla and posterior inferior cerebellum), middle (pons and anterior inferior cerebellum), and distal (rostral brainstem, superior cerebellum), and occipital and temporal lobes. The determination of the classification of a fetal‐type posterior cerebral artery (PCA) as complete or partial was made based on the evaluation of MRA and CTA. CTA was performed using a Neusoft 16‐slice CT scan machine, model Neuviz 16. A complete fetal‐type PCA was identified if the P1 segment was not visible, while a partial fetal‐type PCA was characterized by a smaller P1 segment compared to the posterior communicating artery on brain MRI. BAH was defined as a BA diameter smaller than 2 mm, which was calculated using TOF source images or CTA at the mid‐pons level. Measurements of the V3 segment of the vertebral artery were obtained at its midlevel, while the V4 segments were measured 10 mm cranial to the entrance of the vertebral artery into the foramen magnum. VAH was defined as an internal of both V3 and V4 diameter equal to or less than 2 mm.

Vascular dissection was diagnosed on MRI or CTA when intramural hematoma, intimal flap, pearl‐and‐string, or double‐lumen signs were observed. Neuroradiologists and neurologists reviewed the brain images, with the former being responsible for interpreting the fetal‐type PCA.

### 2.2 Statistical statistics

The data were analyzed using statistical package for social sciences (SPSS) version 22 (IBM Inc.). Data were described using central and dispersion indicators and frequency. Analysis and comparison of quantitative data between groups was done with *t*‐test, analysis of variance, or its non‐parametric equivalents and comparing several qualitative variables with a chi‐square (or Fisher's exact) test. All statistical tests were applied two‐sided. *p* < 0.05 was considered as the level of statistical significance.

## 3 RESULTS

Over the course of the study period from 2016 to 2022, 28,000 brain MRIs were accessible within the records of Ghaem Hospital. Among these, 974 patients were diagnosed with PC ischemic stroke. One hundred fifty‐five eligible patients with an average age of 60.44 ± 13.95 years were included in the study, of which 97 patients (62.6%) were male. Table 1 highlights the demographic characteristics and previous diseases of the patients included in the study.

**Table 1:** Demographic characteristics, history of previous diseases, and characteristics of ischemia in study participants.

Characteristics	Frequency (%)
Gender	
Male	97 (62.6)
Female	58 (37.4)
PMH	
CVA	25 (16.1)
DM	8 (5.2)
HTN	44 (28.4)
IHD	5 (3.2)
DM and HTN	38 (24.5)
DM, HTN, and IHD	10 (6.5)
Location of ischemia	
Proximal	49 (31.6)
Middle	62 (40)
Distal	28 (18.1)
Temporo‐occipital	55 (35.5)
Pattern of infarction	
Right	59 (38.1)
Left	68 (43.9)
Bilateral	8 (5.2)
Multiple	20 (12.9)
VAH	
Right	67 (43.2)
Left	35 (22.6)
Bilateral	5 (3.2)
Fetal origin	
Complete unilateral on the right	38 (24.5)
Complete unilateral on the left	12 (7.7)
Partial unilateral on the right	12 (7.7)
Partial unilateral on the left	6 (3.9)
Complete bilateral	14 (9)
Partial bilateral	8 (5.2)
BAH	6 (3.9)
Dissection	
Right	4 (2.6)
Left	4 (2.6)

Abbreviations: BAH, basilar artery hypoplasia; CVA, cerebrovascular accident; DM, diabetes; HTN, hypertension; IHD, ischemic heart disease; PMH, past medical history; VAH, vertebral artery hypoplasia.

### 3.1 Location of ischemia

The present study analyzed the association between the location of ischemia and the presence of vertebral hypoplasia, basilar hypoplasia, and fetal origin among patients with PC stroke (Table 2). The statistical analysis showed that there were no significant differences between the location of ischemia (categorized as four sites: proximal, middle, distal, and temporo‐occipital) and the presence/absence of VAH (*p* > 0.05). Additionally, the association between the location of ischemia and the status of the BA or the fetal origin was not significantly different (*p* > 0.05 for both).

**Table 2:** The association between the location of ischemia and the presence of VAH, basilar hypoplasia, and fetal origin.

	Location of ischemia			
	Proximal	Middle	Distal	Temporo‐occipital
VAH (*n*, %)				
None	32 (66.7)	31 (64.6)	39 (81.3)	28 (58.3)
Right	44 (65.7)	42 (62.7)	56 (83.6)	43 (64.2)
Left	27 (77.1)	18 (51.4)	28 (80)	24 (68.6)
Bilateral	3 (60)	2 (40)	4 (80)	5 (100)
*p* Value	0.637	0.471	0.971	0.283
VAH (*n*, %)				
None	32 (66.7)	31 (64.6)	39 (81.3)	28 (58.3)
Unilateral	71 (69.6)	60 (58.8)	84 (82.4)	67 (65.7)
Bilateral	3 (60)	2 (40)	4 (80)	5 (100)
*p* Value	0.861	0.519	0.980	0.164
BAH (*n*, %)				
No	102 (68.5)	91 (61.1)	122 (81.9)	94 (63.1)
Yes	4 (66.7)	2 (33.3)	5 (83.3)	6 (100)
*p* Value	>0.999	0.218	0.704	0.090
Fetal origin (*n*, %)				
None	42 (64.6)	39 (60)	54 (83.1)	42 (64.6)
Complete unilateral on the right	26 (68.4)	26 (68.4)	30 (78.9)	25 (65.8)
Complete unilateral on the left	9 (75)	4 (33.3)	11 (91.7)	8 (66.7)
Partial unilateral on the right	9 (75)	7 (58.3)	9 (75)	5 (41.7)
Partial unilateral on the left	3 (50)	5 (83.3)	4 (66.7)	4 (66.7)
Complete bilateral	11 (78.6)	8 (57.1)	12 (85.7)	10 (71.4)
Partial bilateral	6 (75)	4 (50)	7 (87.5)	6 (75)
*p* Value	0.847	0.376	0.846	0.747

Abbreviations: BAH, basilar artery hypoplasia; VAH, vertebral artery hypoplasia.

### 3.2 Pattern of infarction

**Figure 1:**
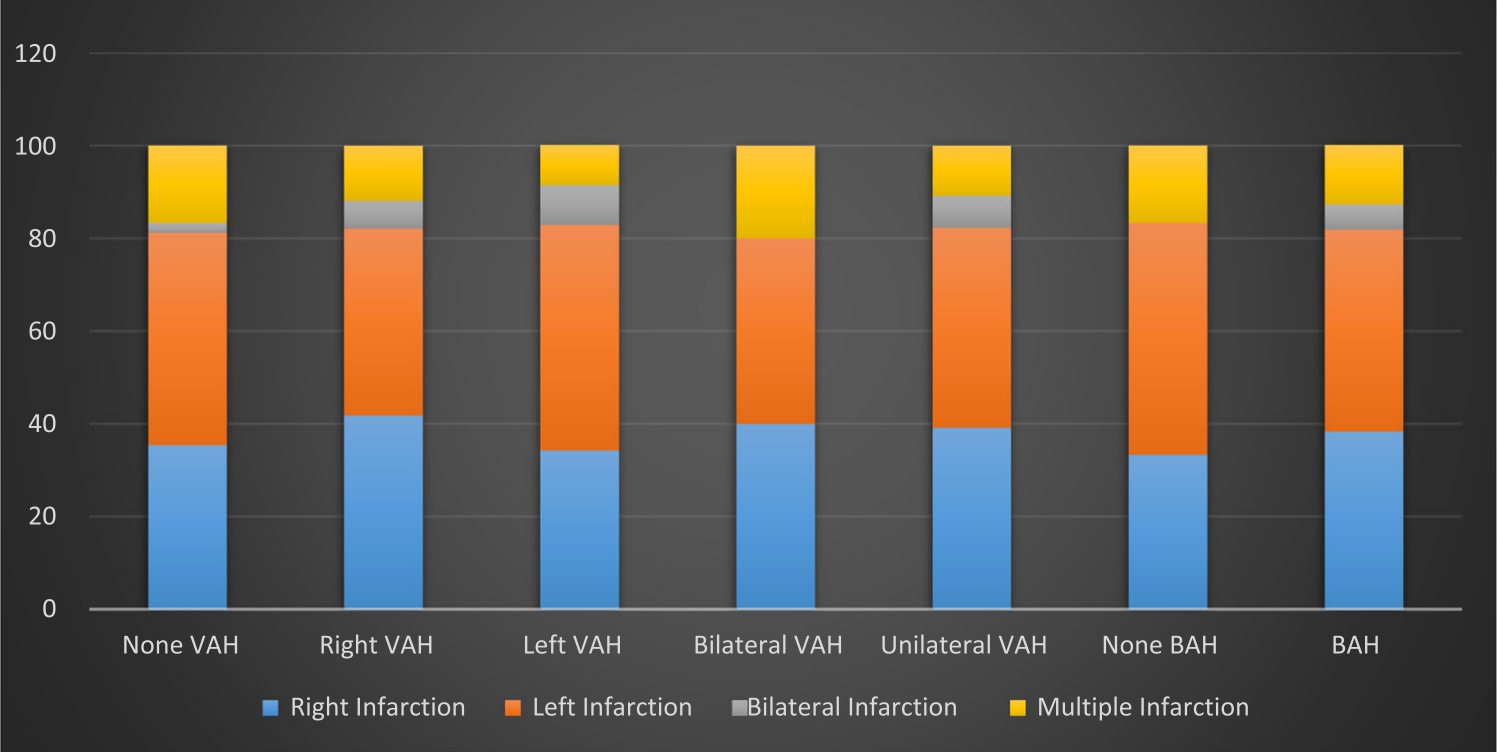
Pattern of ischemia and the presence of VAH and basilar hypoplasia. BAH, basilar artery hypoplasia; VAH, vertebral artery hypoplasia.

The most prevalent infarction pattern in the present study was right infarction with right unilateral VAH; the least common was bilateral infarction with bilateral VAH (Table 3). Figure 1 illustrates the pattern of ischemia and the presence of VAH and basilar hypoplasia in this study. In the present study, the association between the pattern of ischemia and the location or the presence/absence of VAH, basilar hypoplasia, and fetal origin was analyzed, which showed that neither of the latter is significantly different between the four patterns of infarction introduced in this study (*p* > 0.05).

**Table 3:** The association between the pattern of ischemia and the presence of VAH, basilar hypoplasia, and fetal origin.

	Pattern of infarction			
	Right	Left	Bilateral	Multiple
VAH (*n*, %)				
None	17 (35.4)	22 (45.8)	1 (2.1)	8 (16.7)
Right	28 (41.8)	27 (40.3)	4 (6)	8 (11.9)
Left	12 (34.3)	17 (48.6)	3 (8.6)	3 (8.6)
Bilateral	2 (40)	2 (40)	0 (0)	1 (20)
*p* Value	0.898			
VAH (*n*, %)				
None	17 (35.4)	22 (45.8)	1 (2.1)	8 (16.7)
Unilateral	40 (39.2)	44 (43.1)	7 (6.9)	11 (10.8)
Bilateral	2 (40)	2 (40)	0 (0)	1 (20)
*p* Value	0.341			
BAH (*n*, %)				
No	57 (38.3)	65 (43.6)	8 (5.4)	19 (12.8)
Yes	2 (33.3)	3 (50)	0 (0)	1 (16.7)
*p* Value	0.923			
Fetal origin (*n*, %)				
None	25 (38.5)	28 (43.1)	3 (4.6)	9 (13.8)
Complete unilateral on the right	15 (39.5)	17 (44.7)	1 (2.6)	5 (13.2)
Complete unilateral on the left	5 (41.7)	4 (33.3)	2 (16.7)	1 (8.3)
Partial unilateral on the right	1 (8.3)	7 (58.3)	1 (8.3)	3 (25)
Partial unilateral on the left	2 (33.3)	3 (60)	0 (0)	1 (16.7)
Complete bilateral	6 (42.9)	6 (42.9)	1 (7.1)	1 (7.1)
Partial bilateral	5 (62.5)	3 (37.5)	0 (0)	0 (0)
*p* Value	0.794			

Abbreviations: BAH, basilar artery hypoplasia; VAH, vertebral artery hypoplasia.

We also analyzed the data using another classification for the infarction pattern: unilateral, bilateral, or multiple infarctions. However, as evident in Table 4, no significant difference was observed between the location and presence/absence of VAH, BAH, and fetal origin in the three groups introduced for the pattern of infarction (*p* > 0.05).

**Table 4:** The association between the pattern of infarction and the presence of VAH, basilar hypoplasia, and fetal origin.

	Pattern of infarction		
	Unilateral	Bilateral	Multiple
Vertebral hypoplasia (*n*, %)			
None	39 (81.3)	1 (2.1)	8 (16.7)
Right	55 (82.1)	4 (6)	8 (11.9)
Left	29 (82.9)	3 (8.6)	3 (8.6)
Bilateral	4 (80)	0 (0)	1 (20)
*p* Value	0.770		
Vertebral hypoplasia (*n*, %)			
None	39 (81.3)	1 (2.1)	8 (16.7)
Unilateral	84 (82.4)	7 (6.9)	11 (10.8)
Bilateral	4 (80)	0 (0)	1 (20)
*p* Value	0.593		
Basilar hypoplasia (*n*, %)			
No	122 (81.9)	8 (5.4)	19 (12.8)
Yes	5 (83.3)	0 (0)	1 (16.7)
*p* Value	0.822		
Fetal origin (*n*, %)			
None	53 (81.5)	3 (4.6)	9 (13.8)
Complete unilateral on the right	32 (82.4)	1 (2.6)	5 (13.2)
Complete unilateral on the left	9 (75)	2 (16.7)	1 (8.3)
Partial unilateral on the right	8 (66.7)	1 (8.3)	3 (25)
Partial unilateral on the left	5 (83.3)	0 (0)	1 (16.7)
Complete bilateral	12 (85.7)	1 (7.1)	1 (7.1)
Partial bilateral	8 (100)	0 (0)	0 (0)
*p* Value	0.746		

### 3.3 Age and gender

Studying the association between the location of ischemia and age and gender showed that no significant difference was observed between the four categories of ischemia and the patient's age and gender (*p* > 0.05 for all) (Table 5). Similar results were obtained for the pattern of infarction, as no significant difference was observed between the groups in terms of age or gender (*p* > 0.05) (Table 6).

**Table 5:** The association between the location of ischemia and age and gender.

	Location of ischemia			
	Proximal	Middle	Distal	Temporo‐occipital
Age (mean ± SD)				
Positive	61.86 ± 13.34	60.32 ± 14.52	59.72 ± 14.16	59.58 ± 13.41
Negative	57.37 ± 14.87	60.61 ± 13.17	63.68 ± 12.71	62 ± 14.88
*p* Value	0.062	0.900	0.176	0.303
Gender (f, %)				
Female	15 (25.9)	23 (39.7)	13 (22.4)	24 (41.4)
Male	34 (35.1)	39 (40.2)	15 (15.5)	31 (32)
*p* Value	0.285	>0.999	0.289	0.289

**Table 6:** The association between the pattern of infarction and age and gender.

	Pattern of infarction			
	Right	Left	Bilateral	Multiple
Age (mean ± SD)	60.97 ± 14.01	59.60 ± 13.37	57.63 ± 12.55	62.85 ± 16.68
*p* Value	0.745			
Gender (f, %)				
Female	21 (36.2)	25 (43.1)	3 (5.2)	9 (15.5)
Male	38 (39.2)	43 (44.3)	5 (5.2)	11 (11.3)
	0.899			

### 3.4 Basilar hypoplasia

The association between the presence of basilar hypoplasia and different findings was studied in this study. The most common form of VAH present in the case of basilar hypoplasia was bilateral VAH. The statistical analysis showed a significant association between basilar hypoplasia and VAH (*p* < 0.001). Analyzing the association between basilar hypoplasia and different types of fetal origin was statistically significant (*p* < 0.05). We also categorized fetal origin types into “non or unilateral” and “bilateral,” and the comparison showed a significant difference between the two groups in terms of the presence of basilar hypoplasia (*p* < 0.05) (Table 7).

**Table 7:** The association between basilar hypoplasia and the presence of VAH and fetal origin.

	Basilar hypoplasia	
	Yes	No
VAH		
None	0 (0)	48 (100)
Right	1 (1.5)	66 (98.5)
Left	0 (0)	35 (100)
Bilateral	5 (100)	0 (0)
*p* Value	<0.001	
Fetal origin		
None	1 (1.5)	64 (98.5)
Complete unilateral on the right	1 (2.6)	37 (97.4)
Complete unilateral on the left	0 (0)	12 (100)
Partial unilateral on the right	0 (0)	12 (100)
Partial unilateral on the left	0 (0)	6 (100)
Complete bilateral	2 (14.3)	12 (85.7)
Partial bilateral	2 (25)	6 (75)
*p* Value	0.014	
Fetal origin		
None or unilateral	2 (1.5)	131 (98.5)
Bilateral	4 (18.2)	18 (81.8)
*p* Value	0.004	

**Figure 2:**
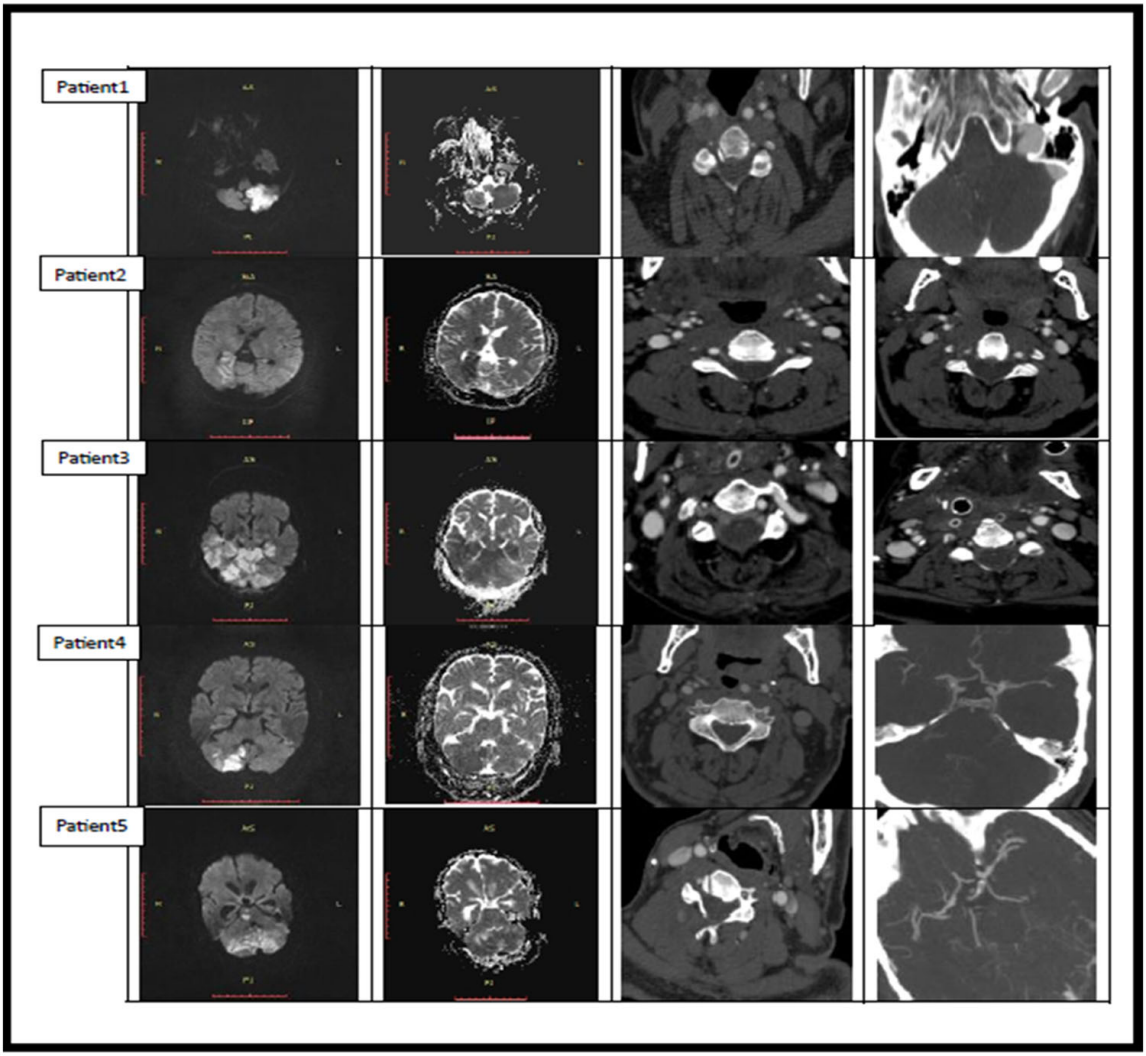
Diffusion‐weighted magnetic resonance imaging, apparent diffusion coefficient, and computed tomography angiography images in the patients of this study show acute infarction, sometimes multiple in the area of posterior cerebral artery blood supply and fetal type variants, hypoplasia of the vertebral arteries and basilar artery, and dissection in the vertebral artery.

Figure 2 illustrates diffusion‐weighted imaging (DWI), apparent diffusion coefficient (ADC), and CT angiography images of the patients in this study, revealing acute infarction, occasionally multiple, within the region of blood supply from the posterior cerebral artery, fetal type variants, hypoplasia of the vertebral arteries and BA, and dissection in the vertebral artery.

## 4 DISCUSSION

The current study evaluated the correlation between VAH and fetal‐type variants of posterior cerebral arteries with regard to the pattern of infarction and the location of ischemia. The findings of this study indicate that the presence of VAH, BAH, and fetal‐type variants of posterior cerebral arteries was not found to be associated with the infarction pattern and location of ischemia. Furthermore, age and sex were not significant determinants of the location and pattern of ischemia in PCA stroke patients. However, this study showed that BAH is associated with fetal origin type and whether VAH is unilateral or bilateral. The occurrence of VAH among patients with PC stroke was 69%, whereas prior research has indicated a range of 10.8%–81.1%^21^, ^22^, ^23^ potentially attributable to variations in ethnicities and research methodologies. In another study in 2021, Dinç et al. reported that 225 out of 609 patients (36.9%) had VAH, which is higher than in our study.^21^ However, the discrepancy may be explained by the fact that all subjects in the present study had a PC stroke, whereas their study included individuals with acute ischemic stroke as the inclusion criteria. Numerous studies have indicated an autonomous function of VAH in the development of regional hypoperfusion in the PC, potentially resulting in ischemic events when accompanied by traditional cerebrovascular accident risk factors.^24^

Ultrasonography and MRA are the primary methods utilized in studies examining the association between PC stroke and VAH.^25^, ^26^, ^27^, ^28^ Although ultrasonography is considered being more accessible and less invasive compared to MRA, it does have certain limitations. For instance, it is unable to accurately identify conditions such as aplasia, hypoplasia, and occlusion.^22^ Given that the present study used MRA, the results could be more reliable than similar studies with ultrasonography. In a study by Chin et al. in 2017, they reported the prevalence of VA at 45%, which is lower than reported in our study and can be explained by the fact that their modality for diagnosis was ultrasonography. The most frequent underlying diseases were hypertension, diabetes, and dyslipidemia, which align with our study.^29^

Many studies have shown that BAH is associated with PC stroke; however, no exact explanation has been offered. Nonetheless, it is believed that BAH predisposes patients to atherosclerosis in the PC, ultimately causing ischemia in this system.^9^, ^30^

A more detailed analysis revealed that fetal origin was evident in almost 60% of the patients, with complete unilateral origin on the right side being the most common type observed, accounting for nearly 25% of the cases. Other types were observed in less than 10% of patients. Previous studies reported the incidence of unilateral and bilateral fetal‐type variants of the PCA artery between 4% and 26% and 2% and 4%, respectively.^31^, ^32^

Similar to other studies, our study possessed both strengths and limitations. A limitation of the present study was its cross‐sectional design and the absence of patient follow‐up, which hindered the investigation of outcome disparities among patients with PCA artery fetal‐type variants and patients with VAH, in comparison to other patients. Conversely, to the best of our knowledge, this study was the first of its kind in the eastern region of the country to explore VAH and PCA fetal‐type variants in patients with ischemic stroke, signifying the innovativeness of our study.

The present study had other limitations including its single‐center approach, the absence of control groups, and the failure to employ biological markers and molecular biology techniques to extensively explore the underlying mechanisms involving VAH, fetal‐type variants of the posterior cerebral artery, and PC stroke.

It is our recommendation that in the years to come, further investigations be carried out to examine the correlation between different forms of VAH and fetal‐type variants of the PCA artery, and their association with PC stroke in patients. It is crucial to assess patient outcomes and observe the recurrence of infarcts. Furthermore, the acquisition of additional data through collaborations between multiple centers will contribute to enhancing the study's reliability and generalizability, thus bolstering the persuasiveness of the findings. it is recommended to also include an appropriate control group, such as healthy individuals or patients without PC stroke. Through the comparison of imaging findings and vascular variations in both the control and patient groups, a more precise evaluation of the link between VAH, fetal‐type variants of the posterior cerebral artery, and PC stroke can be achieved. It is recommended to conduct additional research on the underlying mechanisms linking VAH, fetal‐type variants of the posterior cerebral artery, and PC stroke. This can be achieved through the utilization of biological markers, molecular biology techniques, or animal models. This will facilitate a more comprehensive comprehension of the association between these vascular abnormalities and the onset of stroke.

## 5 CONCLUSION

Hypoplasia of the vertebral artery was observed in over 65% of the patients in the current study, with bilateral hypoplasia seen in 3% of the cases. Fetal origin was also detected in approximately 60% of the patients, with the most prevalent form being one‐sided complete right fetal origin, observed in nearly 25% of the patients. Despite previous research, in our study we found no significant association between VAH and PCA fetal‐type variants with different ischemia locations and infarct patterns. Our conclusion is that VAH and PCA fetal‐type variants are frequently observed in cases of PC stroke, emphasizing the significance of anatomical variants in cerebral blood configuration. Furthermore, there was an absence of definitive evidence suggesting that VAH and fetal origin could be considered predisposing factors for diverse ischemia locations or infarct patterns in PC stroke Thus, it is imperative to conduct additional studies to shed light on this unresolved issue. Furthermore, employing cutting‐edge flow measurement techniques is essential for a more comprehensive evaluation of the impact of VAH and fetal origin on different sites of ischemia and infarct patterns.

## AUTHOR CONTRIBUTIONS

**Parvaneh Layegh**: Conceptualization; supervision; investigation; writing—original draft; project administration; visualization. **Lida Jarahi**: Methodology; software; formal analysis. **Ehsan Hassannejad**: Writing—original draft; methodology; writing—review and editing; data curation; formal analysis. **Marziye Arab**: Writing—original draft; data curation; investigation. **Marziye Arab** had full access to all of the data in this study and takes complete responsibility for the integrity of the data and the accuracy of the data analysis.

## CONFLICT OF INTEREST STATEMENT

The authors declare no conflict of interest.

## ETHICS STATEMENT

The method was approved in compliance with scientific and ethical standards. All methods were performed in line with the relevant guidelines and regulations. The Medical Ethics Committee of Mashhad University of Medical Science approved this study.

## TRANSPARENCY STATEMENT

The lead author Marziye Arab affirms that this manuscript is an honest, accurate, and transparent account of the study being reported; that no important aspects of the study have been omitted; and that any discrepancies from the study as planned (and, if relevant, registered) have been explained.

## Data Availability

The datasets created during the current study are not publicly accessible due to the possibility of compromising the privacy of individuals.
